# Solution-based synthesis of wafer-scale epitaxial BiVO_4_ thin films exhibiting high structural and optoelectronic quality†

**DOI:** 10.1039/d1ta10732a

**Published:** 2022-04-22

**Authors:** Viktoria F. Kunzelmann, Chang-Ming Jiang, Irina Ihrke, Elise Sirotti, Tim Rieth, Alex Henning, Johanna Eichhorn, Ian D. Sharp

**Affiliations:** Walter Schottky Institute and Physics Department, Technische Universität München Am Coulombwall 4 85748 Garching Germany sharp@wsi.tum.de

## Abstract

We demonstrate a facile approach to solution-based synthesis of wafer-scale epitaxial bismuth vanadate (BiVO_4_) thin films by spin-coating on yttria-stabilized zirconia. Epitaxial growth proceeds *via* solid-state transformation of initially formed polycrystalline films, driven by interface energy minimization. The (010)-oriented BiVO_4_ films are smooth and compact, possessing remarkably high structural quality across complete 2′′ wafers. Optical absorption is characterized by a sharp onset with a low sub-band gap response, confirming that the structural order of the films results in correspondingly high optoelectronic quality. This combination of structural and optoelectronic quality enables measurements that reveal a strong optical anisotropy of BiVO_4_, which leads to significantly increased in-plane optical constants near the fundamental band edge that are of particular importance for maximizing light harvesting in semiconductor photoanodes. Temperature-dependent transport measurements confirm a thermally activated hopping barrier of ∼570 meV, consistent with small electron polaron conduction. This simple approach for synthesis of high-quality epitaxial BiVO_4_, without the need for complex deposition equipment, enables a broadly accessible materials base to accelerate research aimed at understanding and optimizing photoelectrochemical energy conversion mechanisms.

## Abstract 2

Epitaxial, (010)-oriented BiVO_4_ films on 2′′ wafers synthesized by spin-coating and metalorganic decomposition exhibit remarkably high crystal quality. Spectroscopy reveals a large uniaxial optical anisotropy, confirming theoretical predictions.

## Graphical abstract

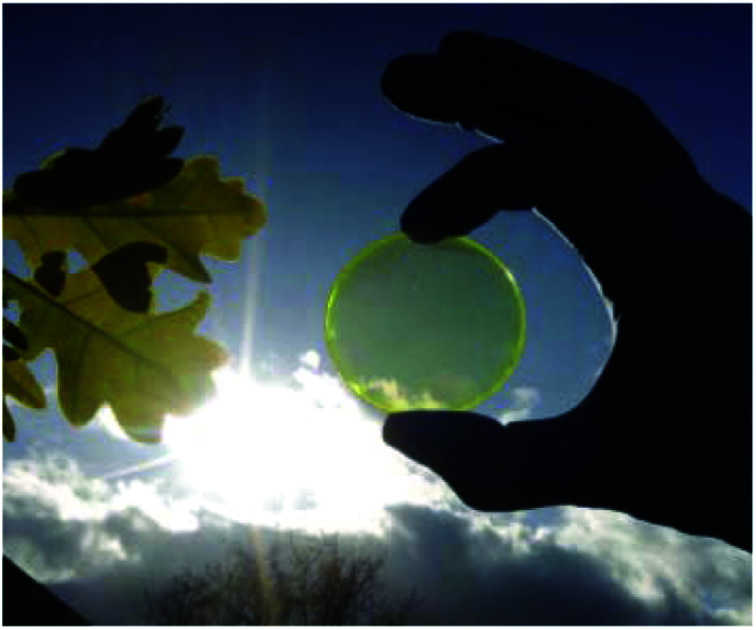

## Introduction

Ternary metal oxide semiconductors have attracted considerable interest as photoelectrode materials for the sustainable conversion of sunlight to chemical fuels. Within this class of compounds, monoclinic scheelite (ms-)bismuth vanadate (BiVO_4_) currently stands as the best performing photoanode material due to its moderate band gap of ∼2.5 eV, favorable band edge energetics, and ability to be stabilized in aqueous solutions.^1–6^ Despite the significant improvements in photoelectrochemical performance characteristics of BiVO_4_ in recent years, critical questions regarding the roles of polaron transport and recombination, bulk and interfacial defects, and surface chemical transformations on light-to-chemical energy conversion remain unanswered. With a few notable exceptions, most studies of BiVO_4_ have focused on polycrystalline or nanostructured thin films, the internal complexities of which have hindered mechanistic elucidation of these characteristics. Although additional insights have been enabled by studies of highly oriented thin films,^7,8^ minimization of structural disorder is necessary to enable better understanding of critical loss processes. To address this knowledge gap, epitaxial films can serve as important model systems. Until now, epitaxial BiVO_4_ films have primarily been synthesized *via* pulsed laser deposition (PLD),^9–14^ though molecular beam epitaxy (MBE)^15^ and chemical vapor deposition (CVD)^16^ processes have also been reported. While studies based on such samples have provided impactful insights into charge transport,^17^ energetics,^18^ and surface reactivity,^19^ these deposition methods require sophisticated experimental infrastructure and complex parameter optimization. As a consequence, only a handful of groups have succeeded in achieving heteroepitaxial thin films and, to the best of our knowledge, wafer-scale growth of high structural quality epitaxial BiVO_4_ by any method has not yet been reported. Thus, realization of scalable solution-based approaches for epitaxial growth are urgently needed to accelerate mechanistic understanding and rational development of materials for photoelectrochemical energy conversion.

In this work, we introduce a new approach for wafer-scale synthesis of epitaxial ms-BiVO_4_ on yttria-stabilized zirconia (YSZ) (001) using a solution-based process comprising spin-coating, metalorganic decomposition, and solid-state epitaxial transformation. This technique allows facile fabrication of thin films possessing a high optoelectronic quality and a structural quality that exceeds previously reported epitaxial BiVO_4_ films produced using much more sophisticated deposition approaches, as exemplified by high-resolution X-ray diffraction (HR-XRD) measurements. We find that, compared to polycrystalline thin films, (010) BiVO_4_ is characterized by significantly increased band-edge optical constants due to the strong uniaxial anisotropy of the material, indicating the importance of orientation control on maximizing light absorption in photoelectrodes. More generally, this facile and scalable method for synthesizing epitaxial BiVO_4_ will enable widespread availability of highly ordered material required for elucidating physical and chemical mechanisms of photoelectrochemical energy conversion.

## Experimental section

### Synthesis procedure for BiVO_4_ thin films

Prior to deposition of BiVO_4_/YSZ, the two-inch (2′′) YSZ (001) wafer (8 mol% yttria, undoped, Alineason) was prepared by careful rubbing of the surface in acetone with clean room quality cleaning swabs, followed by cleaning in an ultrasonic bath, first for 10 min in acetone and subsequently for 10 min in isopropanol. The sample substrate was then dried under flowing N_2_. Afterwards, the YSZ wafer was placed on a 1 mm thick steel plate and pre-annealed at 500 °C for 30 min in air at ambient pressure in a muffle furnace (LT 3/12, Nabertherm). This pre-annealing step was found to increase the wettability of the BiVO_4_ precursor solution on the substrate, which enabled high homogeneity and coverage over the full 2′′ wafer scale. The contact angle (CA) measurements of the precursor solution on YSZ in Fig. S1† confirm the reduction of the CA from ∼9° for as-cleaned YSZ to an undeterminably low CA for the pre-annealed YSZ. This shows the immediate, uniform distribution of the precursor solution on the annealed YSZ surface caused by an improved wettability due to the annealing treatment, which is beneficial for the growth of a continuous BiVO_4_ layer.

The BiVO_4_ precursor solution was prepared in a 1 : 1 ratio from two separate solutions, one containing 0.2 M bismuth(iii) nitrate pentahydrate (Bi(NO_3_)_3_·5H_2_O, Sigma-Aldrich) in acetylacetone (C_5_H_8_O_2_, Sigma-Aldrich) and the other containing 0.03 M vanadium(iv)-oxy acetylacetonate (OV(C_5_H_7_O_2_)_2_, Sigma Aldrich) in acetylacetone. Each solution was sonicated for 10 min, after which the solutions were mixed and the final precursor solution was sonicated for an additional 5 min. Finally, the prepared solution was filtered with a PET membrane filter (0.2 μm pore size, 15 mm diameter, Carl Roth) to ensure a clean and fully mixed precursor. A drop of 500 μl precursor solution was spin-coated on a YSZ wafer with an acceleration rate of 150 rpm s^−1^, a spin speed of 1000 rpm for 12 s, and a deceleration rate of 150 rpm s^−1^. Subsequently, the wafer was placed on a 1 mm thick steel plate and annealed at 500 °C for 10 min in air at ambient pressure in a muffle furnace (LT 3/12, Nabertherm). After annealing, the wafer was taken out of the oven to cool down to room temperature for 12 min. Repeating this growth sequence for nine layers resulted in an approximately 43 nm thick polycrystalline (poly-)BiVO_4_/YSZ thin film. To achieve an epitaxial, single-phase BiVO_4_ layer, the resulting film was annealed at 650 °C for 10 min in a confined air volume of approximately 600 mm^3^. The volume confinement in the muffle furnace was formed by placing a clean, thermally stable silicon wafer approximately 300 μm above the BiVO_4_/YSZ wafer, supported by other wafer pieces that still allowed external air to enter. The purpose of this confinement was to mitigate loss of volatile elements from the surface during the annealing at 650 °C, thereby enabling a stoichiometric film to be retained. This configuration may have also reduced convective air flow over the surface, thereby improving temperature uniformity during the annealing treatment.

For reference measurements of polycrystalline BiVO_4_ on fused silica, wafers of fused silica (T24003, Siegert Wafer) with a diameter of 10 cm were rinsed with isopropanol and carefully scrubbed with a cleaning swab in a detergent solution (Alconox, Alconox Ing.). After rinsing with deionized water, the wafer was treated in an ozone cleaner for 10 min (UVC-1014, NanoBioAnalytics). The precursor preparation and spin-coating procedure were similar to the ones described above for the BiVO_4_/YSZ films, except that 1 ml precursor solution per layer was used, the wafer was placed on a 1 mm thick aluminum plate, and a final 2 h anneal was performed at 500 °C, as previously reported in the literature.^20^

### Material characterization

Crystallographic characterization was performed using an X-ray diffractometer (SmartLab, Rigaku) equipped with a Cu anode and a 2-bounce Ge (220) monochromator. Out-of-plane *θ*–2*θ* scans were done between 2*θ* = 28–37° with 0.005° steps, with 1.0 mm incident/receiving slit sizes in the longitudinal direction. The rocking curves of the BiVO_4_ (040) reflection were measured with reduced 0.2 mm slit sizes to achieve better resolution. Azimuthal φ-scans were performed in asymmetric geometry: for the YSZ (204) reflection the incident and diffraction angles were respectively set as 68.67° and 15.54° relative to the sample surface (2*θ* = 84.21°). For the BiVO_4_ (082) reflection the incident and diffraction angles were 81.12° and 3.72°, respectively (2*θ* = 84.84°). Two-dimensional reciprocal space maps were collected with a point-like, non-monochromatic Cu X-ray source and a CCD detector (HyPix-3000, Rigaku) without any receiving optics; the sample tilting angle *χ* was varied between −5° and 70°. For grazing-incidence X-ray diffraction, no monochromator was used and the X-ray incident angle was fixed at 0.5°. The 2*θ* angle was then scanned between 10° and 70°. For the thickness determination of the epitaxial (epi-)BiVO_4_/YSZ, the interference oscillations around the BiVO_4_ (040) reflection were fit with a pseudo-Voigt function with a constant background (Fig. S2b†).

Raman spectra were obtained with a home-built setup with a 532 nm wavelength laser (Torus 532, Laser Quantum). Prior to measurement, the system was calibrated to the 520 cm^−1^ peak of a single-crystal silicon reference. The backscattered light was analyzed with a spectrometer (iHR5500, HORIBA Scientific) equipped with a 2400 mm^−1^ grating and a CCD detector (Symphony II, HORIBA Scientific).

X-ray photoelectron spectroscopy data were recorded with a Kratos Axis Ultra setup equipped with a monochromatic Al Kα X-ray source and an applied power of 225 W. To avoid surface charging, charge neutralization was performed with the Kratos Axis electron charge neutralization system with an applied filament current of 0.45 A, a filament bias voltage of 1 V, and a charge balance voltage of 3 V. The survey spectra were measured with a pass energy of 160 eV and a step size of 1 eV, and the core level spectra were obtained with a pass energy of 10 eV and a step size of 0.03 eV. Remaining charging artifacts prevent detailed spectral fitting of individual components.

For the investigation of the bulk composition of the epitaxial BiVO_4_ films, a Bruker XFlash6130 energy-dispersive X-ray spectrometer, mounted in a Zeiss EVO MA10 for sample alignment imaging, was used. The EDX detection spot size was 1 μm and an electron beam energy of 20 keV was applied. The resulting spectra were analyzed using series fit deconvolution and the Phi-Rho-Z quantification mode.

A Bruker MultiMode 8 atomic force microscope was used for the topography measurements. All measurements were acquired in tapping mode with NSG30 tips (NT-MDT). The images were post-processed with the Bruker Nanoscope Analysis software with 0^th^ order flattening and a 3^rd^ order plane fit to account for instrumental aberrations. Prior to recording the high-resolution and cross-sectional scanning electron microscopy (SEM) images, a ∼3 nm thin carbon coating was sputtered on the investigated BiVO_4_ films to reduce charging. For the cross-sectional SEM images, the samples were then freshly cleaved to obtain a sharp edge. The SEM images were obtained with an NVision 40 FIB-SEM from Carl Zeiss using the secondary electron detector and an electron beam acceleration voltage of 2.0 kV.

### Characterization of optical properties

The absorption coefficient was measured as a function of the photon energy with a home-built photo-thermal deflection setup. In Fig. S3† Tauc analysis was performed on the recorded absorption data to determine the indirect and direct band gap of the epi-BiVO_4_/YSZ and the BiVO_4_/fused silica. The optical transitions can be described by the following equation:(α*hν*)^*n*^ = *A*(*hν* − *E*_g_)where *α* is the measured absorption coefficient, *hν* is the photon energy, *A* is a proportionality factor, and *E*_g_ is the band gap.^21^ The parameter *n* depends on the nature of the transition, with *n* = 1/2 for indirect transitions and *n* = 2 for direct transitions.

The optical constants of the BiVO_4_ films on YSZ and on fused silica were measured with a variable-angle spectroscopic ellipsometer from J. A. Wollam under a light incidence angle of 55°, 60° and 65°. The refractive index, *n*, the extinction coefficient, *κ* (Fig. S4a and c†), and the real, *ε*_1_, and imaginary, *ε*_2_, parts of the dielectric constant (Fig. S4b and d†) were obtained by fitting the spectroscopic ellipsometry data with a layer model developed with the CompleteEASE software from J. A. Wollam. The YSZ substrate was fit with a B-spline model with a fixed thickness of 0.5 mm and a Bruggeman effective medium approximation surface layer accounting for surface roughness. The B-spline layer was then parametrized as a general oscillator (GenOsc) layer consisting of a Cody–Lorentz oscillator including an Urbach absorption term to model absorption at energies below *E*_g_, a Drude oscillator describing free carrier effects on the dielectric response, and a Gaussian oscillator, all fulfilling Kramers–Kronig consistency. For the fused silica substrate, the model as described by Philipp^22^ and found in the CompleteEASE database was used. The BiVO_4_ film was modelled as an additional B-spline layer on the YSZ substrate which was parametrized by a GenOsc layer with a three oscillator model as described by Cooper *et al.*,^6^ with: a Cody–Lorentz oscillator for modelling the indirect band gap of BiVO_4_, including parameters describing the Urbach tail associated with sub-band gap absorption induced by disorder, a PSemi-M0 oscillator for modeling the direct electronic transition in the semiconductor, and a PSemi-Tri oscillator to describe the remaining UV absorption.^6^

### Solid-state conductivity measurements

For the in-plane DC conductivity measurements, Ti/Au (20/80 nm) interdigitated electrodes with a 10 μm spacing were evaporated on the BiVO_4_ surface with e-beam evaporation. The spacing of the interdigitated electrodes and the applied voltage were chosen so that the applied electric field |*E⃑*| was within the low field approximation where the distortion caused by |*E⃑*| is small in comparison to the transport barrier *E*_*σ*_: |*E⃑*| ≪ *E_σ_*/*e* × *d*. Previous studies showed that polaron hopping is predicted to favorably occur between vanadium atoms^23^ so we overestimate the polaron hopping distance between two vanadium ions with *d* = 10 Å. Together with the assumption of a transport barrier of *E*_*σ*_ = 500 meV, as previously reported for undoped BiVO_4_,^11^ we obtain for the electric field: |*E⃑*| ≪ 500 V μm^−1^.

## Results

### Solution-based synthesis approach for epitaxial BiVO_4_

In this study, we choose the conventional unit cell definition for ms-BiVO_4_ with a unique *b*-axis (*a* = 7.247 Å, *b* = 11.697 Å, *c* = 5.090 Å, *β* = 134.225°, Fig. S5†).^24^ Single crystal YSZ (001) was selected as the substrate since its cubic 5.145 Å lattice constant is well matched to the 5.094 Å and 5.196 Å V–V spacing in the BiVO_4_ (010) lattice planes to within 1%.^25^ We note that this YSZ substrate offers several advantages for fundamental studies of energy conversion processes. The transparent and insulating character enable precise characterization of optical properties and charge transport characteristics of the semiconductor absorber, respectively. Furthermore, YSZ is compatible with epitaxial growth of tin-doped indium oxide (ITO), which can eventually serve as an interfacial conductive back contact to epitaxial BiVO_4_.

**Fig. 1:**
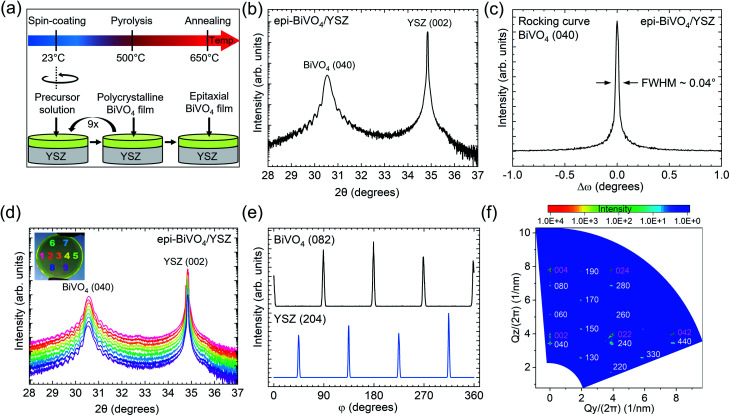
Illustration of synthesis procedure and structural characterization of epitaxial BiVO_4_/YSZ. (a) Schematic of the solution-based synthesis process of epi-BiVO_4_ on a complete 2′′ YSZ wafer, starting with spin-coating, followed by metalorganic decomposition to form a polycrystalline film, and a final annealing step at 650 °C to trigger the solid-state transformation into an epitaxial film. (b) High-resolution XRD *θ*–2*θ* scan of epi-BiVO_4_/YSZ. (c) Rocking curve recorded around the BiVO_4_ (040) peak at 2*θ* = 30.55°. (d) *θ*–2*θ* XRD scans of epi-BiVO_4_/YSZ at different measurement positions on the wafer (inset photo). (e) Azimuthal φ-scans of the epi-BiVO_4_ along the out-of-plane BiVO_4_ (082) and the YSZ (204) direction confirm the epitaxial relationship between the BiVO_4_ and the YSZ. (f) 2D reciprocal space map of the epi-BiVO_4_, with BiVO_4_ and YSZ peaks labelled in red and white, respectively.

Cleaning and thermal pre-treatment of the YSZ wafer were performed to achieve high wettability, coverage, and homogeneity during the spin-coating process (Fig. S1†). The synthetic route to epi-BiVO_4_ layers (for complete details see Methods) is similar to the metalorganic decomposition method that has been previously used for fabrication of polycrystalline material.^20,26^ In brief, a precursor solution was mixed from two solutions containing 0.2 M bismuth(iii) nitrate pentahydrate and 0.03 M vanadium(iv)-oxy acetylacetonate, each dissolved in acetylacetone. As shown in Fig. 1a, the precursor solution was then spin-coated onto a two-inch (2′′) YSZ (001) wafer and subsequently pyrolyzed at 500 °C for 10 min in a muffle furnace in ambient air. Repeating this growth sequence for nine layers resulted in an approximately 43 nm thick BiVO_4_ film with a polycrystalline structure, which was confirmed by grazing-incidence XRD (GI-XRD) (Fig. S2a†). To transform the polycrystalline films into epi-BiVO_4_, we performed a final annealing treatment at 650 °C for 10 min in ambient air. Raman spectroscopy confirms that the films comprise the monoclinic scheelite polymorph of BiVO_4_ both before and after this annealing treatment (Fig. S6†), as indicated by the (VO_4_)^3−^*ν*_1_ symmetric stretching mode at ∼826 cm^−1^.^27^

We note that this last annealing step is performed at a much higher temperature and for a shorter time than for the typical metalorganic decomposition approach for forming poly-BiVO_4_, which is usually carried out at 450–500 °C for several hours. This short time, high-temperature step is crucial for inducing a solid-state transformation of poly-BiVO_4_ into an epitaxial thin film, which will be discussed in detail below. We note that an advantage of this two-step method is that the epitaxial transformation is independent of the specific chemical route used to prepare the initial polycrystalline layer. Therefore, we expect this process to be broadly compatible with the various precursor solution compositions and solvents that have been reported for wet chemical synthesis of polycrystalline BiVO_4_ films.

At the high annealing temperature of 650 °C, it is known that vanadium becomes volatile in air.^28^ Therefore, this step was performed in a confined volume, which helped to maintain the desired stoichiometry. Consistent with prior reports of metalorganic decomposition-derived BiVO_4_,^20,29^ X-ray photoelectron spectroscopy (XPS) reveals a Bi-rich surface (Fig. S7†). The surface Bi : V ratio is found to be ∼1.7 for both the poly- and epi-BiVO_4_ films on YSZ, indicating that the combination of short time and spatial confinement during the 650 °C annealing step suppresses further volatilization of vanadium. Investigation of the bulk composition with energy-dispersive X-ray spectroscopy (EDX) revealed an atomic fraction of ∼48.4 at% bismuth and ∼51.6 at% vanadium (Fig. S8†). This bulk composition is consistent with the results of previous studies on similarly prepared polycrystalline thin films^20^ and confirms the 1 : 1 ratio between Bi and V in BiVO_4_ to within the few percent uncertainty of the EDX measurement.

### Analysis of crystal structure and orientation

The crystallographic orientation and structural quality of the epi-BiVO_4_ films were analyzed using several XRD modes. Combining HR-XRD *θ*–2*θ* scans (Fig. 1b) and grazing-incidence 2*θ* scans (Fig. S2a†) indicates that the final 650 °C annealing step results in a transformation of the starting polycrystalline film into an epi-BiVO_4_ (010) film. Interference fringes surrounding the BiVO_4_ (040) Bragg peak arise from the high homogeneity and planarity of the film, with a period corresponding to a film thickness of ∼42.9 nm (Fig. S2b†). The observation of BiVO_4_ (020) and (060) reflections in extended-range *θ*–2*θ* scans (Fig. S2c†) confirms the monoclinic scheelite structure instead of the tetragonal scheelite phase. The structural quality of the epi-BiVO_4_ film was further quantified by analyzing the rocking curve of the BiVO_4_ (040) reflection at 30.55° (Fig. 1c). Remarkably, the rocking curve full width at half maximum (FWHM) of 0.04° is much lower compared to previous reports on epi-BiVO_4_ films grown on YSZ by PLD (0.097–0.3°)^9–13^ or CVD (0.13°).^16^ Importantly, this indicates that the facile spin-coating method reported here yields epi-BiVO_4_ films with a structural quality comparable to or exceeding that of material deposited by much more sophisticated systems, thereby providing a route to greatly increase availability of epitaxial layers within the research community.

A key advantage of spin-coating is its potential for wafer-scale growth. To assess the growth homogeneity, a systematic series of *θ*–2*θ* scans was performed across the entire 2′′ YSZ wafer. At each measurement position, comparable diffractograms were observed (Fig. 1d) and the FWHMs of the corresponding BiVO_4_ (040) rocking curves are between 0.035° and 0.05° (Fig. S2d†). These measurements confirm the successful growth of a homogeneous, high-quality epi-BiVO_4_ (010) film over the entire surface of the YSZ (001) wafer and could be reliably reproduced in several epi-BiVO_4_/YSZ wafer growth runs (Fig. S9†). In addition, we performed ellipsometry measurements across the diameter of the epitaxial BiVO_4_ film, confirming a uniform thickness of approximately 43 nm (Fig. S10†). Edge effects that are common to spin-coating resulted in film thickening, but were limited to a 4 mm width around the circumference of the wafer.

The in-plane epitaxial relationship between BiVO_4_ and the YSZ substrate is revealed by azimuthal φ-scans of YSZ (204) and BiVO_4_ (082) reflections. As shown in Fig. 1e, both reflections exhibit a 4-fold symmetry and are offset by a ∼45° angle. Given the BiVO_4_ unit cell definition with a unique *b*-axis adopted in this work, its (082) reflection is expected to have a 2-fold symmetry. Therefore, the observed 4-fold symmetry is indicative of 90° in-plane twinning, with domains characterized by in-plane relationships of BiVO_4_ [001] ‖ YSZ [100] and BiVO_4_ [001] ‖ YSZ [010] (Fig. S11†). This assignment is further supported by 2D reciprocal space maps (RSM) with X-rays incident along the YSZ [100] direction (Fig. 1f). Not only does the pattern match with the simulated BiVO_4_ [001](010) ‖ YSZ [100](001) model (Fig. S12a†), but the (330), (440), and (260) BiVO_4_ reflections each exhibit a doublet feature caused by the 90° in-plane twinning. Similar conclusions can be drawn from the RSMs with X-rays incident along the YSZ [110] and [010] directions, as shown in Fig. S12b and c.†

Here, it is important to recognize that BiVO_4_ adopts the more symmetric tetragonal scheelite structure at the temperature used for the conversion of polycrystalline to epitaxial films and undergoes a second-order ferroelastic transformation to the monoclinic structure upon cooling.^30,31^ Therefore, azimuthal twinning occurs during cooling, after the out-of-plane epitaxial relationship has been established, and multi-domain films are commonly observed for epitaxial BiVO_4_ (010) on YSZ.^9,15,32^ Although single domain epitaxial BiVO_4_ (010) films have been claimed by a few groups,^10–12,14^ the experimental evidence for single domain films remains limited.

### Elucidating the solid-state epitaxial transformation mechanism

**Fig. 2:**
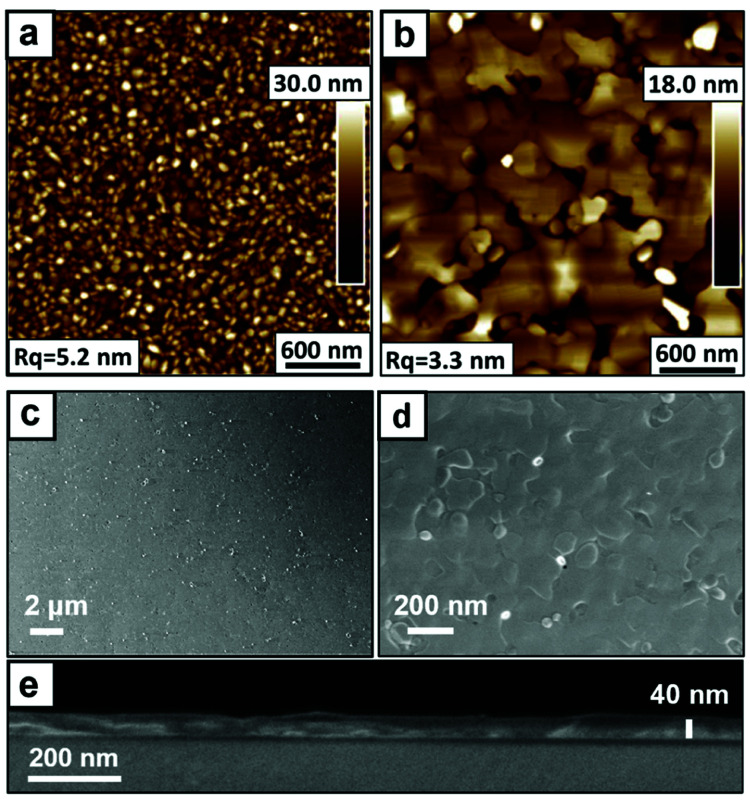
Morphological analysis of BiVO_4_/YSZ before and after the solid-state transformation. AFM topography images of (a) poly-BiVO_4_/YSZ starting material and (b) epi-BiVO_4_/YSZ after annealing at 650 °C. (c and d) Top-view SEM and (e) cross-sectional SEM images of epi-BiVO_4_/YSZ.

To gain further insights into the solid-state epitaxial transformation and characteristics of the synthesized films, the surface morphologies of BiVO_4_ on YSZ were determined by atomic force microscopy (AFM) and scanning electron microscopy (SEM). Prior to the final high-temperature treatment, the polycrystalline film is characterized by a fine grain structure with a root mean square (rms) roughness of 5.2 nm (Fig. 2a). Annealing at 650 °C leads to the emergence of flat terraces with a size ranging from several hundred nm up to few μm, reducing the rms roughness to 3.3 nm (Fig. 2b). Over larger scales, plan view SEM images confirm that the BiVO_4_ layer is smooth and closed, as shown in Fig. 2c and d. This homogeneous coverage is confirmed by XPS measurements, in which no Zr or Y core level peaks from the substrate are detected (Fig. S7†). Cross-sectional SEM images (Fig. 2d) show an approximately uniformly thick and compact BiVO_4_ thin film with an average thickness of 42.8 nm. We note that epitaxial growth of compact BiVO_4_ films on YSZ can be achieved by PLD, but is challenging since islands usually form,^9,10,13^ most likely driven by the unknown energetics of the tetragonal scheelite BiVO_4_/YSZ interface during growth. In the present work, we also observe the formation of small particles when a single pyrolysis step is performed at 650 °C (Fig. S13a†). By contrast, a smoother polycrystalline layer forms at the optimized pyrolysis temperature of 500 °C (Fig. S13b†). Therefore, the two-step process is optimized to ensure compact and planar epitaxial films, while suppressing island formation.

Both AFM and SEM images reveal the presence of scattered particles, which is consistent with a small quantity of remnant mis-oriented grains detected by GI-XRD (Fig. S2a†). The occurrence of these grains points to the mechanism of the solid-state transformation from polycrystalline to epitaxial films, as described in the following. After the spin-coating and 500 °C pyrolysis steps, pole figure measurements indicate a polycrystalline but textured BiVO_4_ film with preferred (010) orientation (Fig. S14†). Such film characteristics are consistent with parallel heterogeneous and homogeneous nucleation events occurring during pyrolysis.^33^ In particular, it has been shown that moderate thermal ramp rates, such as those used here (Fig. S15†), promote nucleation over a broad range of relatively low temperatures, where driving forces for both homogeneous and heterogeneous nucleation are large, resulting in multiple grain configurations.^33,34^ Subsequent high-temperature annealing (650 °C) induces epitaxial grain coarsening *via* the consumption of mis-oriented grains, energetically driven by interface energy minimization and kinetically enabled by high diffusivities.^35^ Although the fine-grained morphology of the original polycrystalline film facilitates rapid grain boundary motion and epitaxial film formation, the driving force for this transformation decreases with decreasing fraction and size of mis-oriented grains.^35^ As described by Queraltò *et al.*, such a mechanism leads to a small and scattered fraction of remnant polycrystalline grains on the surface, as observed in the present work.^35^ A viable strategy for eliminating such grains would be to use rapid thermal annealing during the pyrolysis step to increase the onset temperature of nucleation, which would favor preferred heterogeneous nucleation.

Consistent with this interpretation, we find that 10 min annealing at a lower temperature of 625 °C leads to an incomplete transformation, resulting in mixed domains of polycrystalline and epitaxial material (Fig. S16a†). In contrast, annealing at a higher temperature of 675 °C yields epitaxial films, though local pitting is observed (Fig. S16b†), the precise origin of which is not yet clear. Thus, 650 °C was chosen as the optimal annealing temperature for forming closed epitaxial films.

### Study of optical absorption and anisotropic optical constants of epitaxial BiVO_4_

**Fig. 3:**
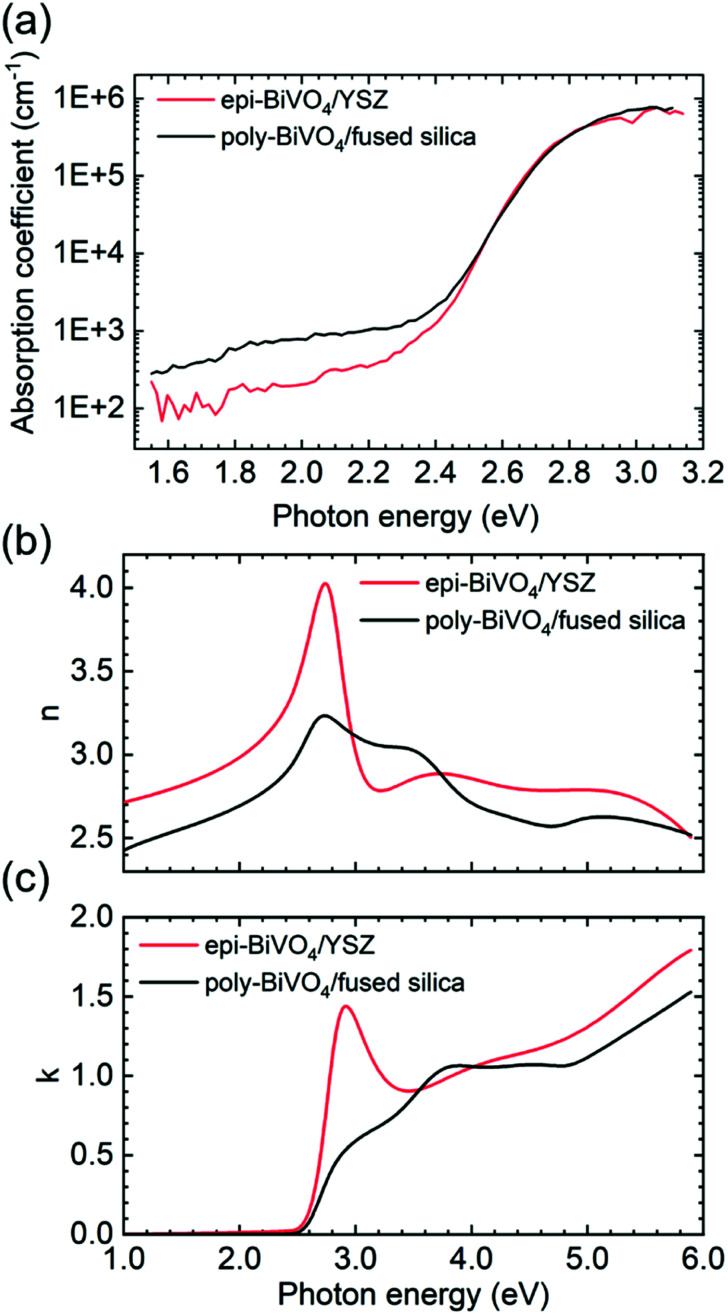
Optical absorption characteristics of epi-BiVO_4_/YSZ and poly-BiVO_4_/fused silica. (a) Absorption spectra of epi-BiVO_4_/YSZ and poly-BiVO_4_/fused silica measured by PDS. (b) The refractive index, *n*, and (c) the extinction coefficient, *κ*, of epi-BiVO_4_/YSZ compared to poly-BiVO_4_/fused silica with randomly oriented grains. Optical constants were obtained by fitting of variable-angle spectroscopic ellipsometry spectra. The strong optical anisotropy of BiVO_4_, which favours excitations with the electric field *E* ‖ [100] and *E* ‖ [001] leads to a significant increase of the optical constants near the band edge for the case of epitaxial [010] BiVO_4_.

We now turn to the optical quality of the films, which was assessed by photothermal deflection spectroscopy (PDS), a highly sensitive method for measuring the absorption coefficient. Fig. 3a shows a comparison of optical absorption spectra from epi-BiVO_4_/YSZ and poly-BiVO_4_/fused silica measured by PDS.

For all films, Tauc analysis yields an indirect band gap of 2.53 eV and a direct transition near 2.72 eV (Fig. S3†). Although slightly smaller direct band gaps have been previously reported for some epi-BiVO_4_ films,^9,12,14–16^ we find no significant difference between poly- and epi-films. Low and nearly identical Urbach energies are observed for all samples, with values of 65 meV for polycrystalline films on fused silica, 62 meV for the textured polycrystalline, and 58 meV for the epitaxial films on YSZ. However, epitaxial material exhibits reduced sub-band gap optical absorption relative to polycrystalline material, indicating moderate reduction of midgap states in the material. The overall optical quality of all films produced *via* metalorganic decomposition is found to be high, comparing favourably with prior reports^6,36^.

The availability of planar and compact epitaxial films offers the intriguing possibility to also investigate anisotropic material properties. Of particular relevance for solar energy conversion is the strong optical anisotropy of BiVO_4_, which has been computationally predicted^37–39^ but only partially investigated experimentally.^31,40^ Here, we performed variable-angle spectroscopic ellipsometry (VASE) to quantify the optical constants and compare results from epitaxial films on YSZ with polycrystalline material possessing random grain orientations. We note that a true anisotropic model cannot be reliably established with such a thin film given the weak contribution from out-of-plane oscillators and the large number of fitting parameters, which lead to non-physical parameter correlation. Therefore, in the present work we approximate the system using an isotropic general oscillator model to extract the optical constants of epi-BiVO_4_ and compare it to poly-BiVO_4_ with randomly oriented grains, as shown in Fig. 3b and c. The corresponding real and imaginary components of the dielectric function are presented in Fig. S4† and a detailed description of the optical model can be found in the Methods section. Importantly, we find a strong enhancement of the optical constants for the electric field *E* ‖ [100] and *E* ‖ [001] compared to *E* ‖ [010]. This result is consistent with prior DFT calculations, which predict band edge O 2p → V 3d dipole excitations associated with the fundamental band gap to be forbidden along the *b*-axis, but allowed along the *a*- and *c*-axes.^38^ To the best of our knowledge, this increase of both optical constants near the band edge due to anisotropy has not been previously reported. This significant increase of the extinction coefficient at the band edge for epi-BiVO_4_ (010) compared to polycrystalline material suggests a preferred BiVO_4_ (010) orientation for efficient light harvesting. Furthermore, this observation explains the different spectral shapes for band edge absorption that have been reported in the literature, which at times include a shoulder or peaked shape near 2.8 eV; films with different texture will naturally exhibit different above-gap absorption spectra due to relative sampling of the anisotropic dielectric function. This conclusion is supported by VASE measurements of textured poly-BiVO_4_/YSZ, which exhibit intermediate optical constants between ideally oriented epi-BiVO_4_ and randomly oriented poly-BiVO_4_/fused silica (Fig. S4†).

### Characterization of electronic charge transport

**Fig. 4:**
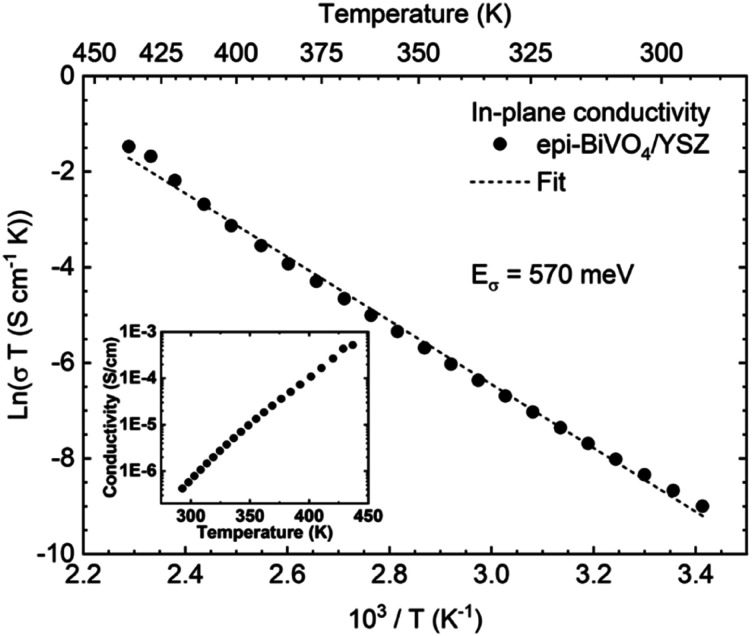
Temperature-dependent in-plane conductivity of epi-BiVO_4_/YSZ. Conductivity, *σ*, plotted as ln(*σT*) *vs.* 10^3^/*T*. The dashed line represents the fit to eqn (1), yielding a charge transport activation energy, *E*_*σ*_, of 570 meV. In the inset, the conductivity is plotted against the temperature in a semi-log plot.

In addition to optical characteristics, electronic transport properties are key for photoelectrochemical energy conversion, as well as for validating the quality of produced epitaxial films. Enabled by the high structural quality of continuous epi-BiVO_4_ films, we measured the in-plane DC conductivity, *σ*, as a function of the temperature, *T*. The resulting temperature-dependent conductivity is consistent with a previously proposed thermally-activated polaron hopping process that follows an approximately linear behavior when plotted as ln(*σT*) *vs.* 1/*T*, as shown in Fig. 4. Fitting of the data (dashed line, Fig. 4) was performed according to1
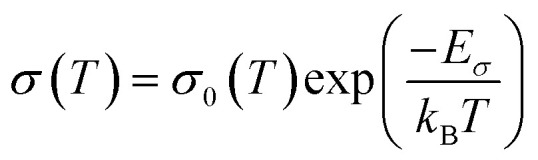
where *σ*_0_(*T*) is a temperature-dependent prefactor, *E*_*σ*_ is the charge transport activation energy, and *k*_B_ the Boltzmann constant.^41,42^

The resulting charge transport activation barrier, *E*_*σ*_, of 570 meV and the absolute conductivity (inset Fig. 4) are in excellent agreement with the previously reported values obtained from undoped BiVO_4_ on YSZ deposited *via* PLD.^11^ Interestingly, the films deposited by Zhang and co-workers were reported to comprise a single domain, whereas our films are characterized by twinning with domain boundaries. The finding of nearly identical transport barriers for both types of films suggests that in-plane transport is not significantly impacted by the presence of domain boundaries. This is surprising given that domain boundaries for epi-BiVO_4_ on indium-doped tin oxide (ITO) were found to act as high-conductivity channels for out-of-plane electrical transport,^11^ pointing to the need for improved understanding of planar defects in this material. Nevertheless, the electrical quality of the films is found to be commensurate with state-of-the-art material fabricated by PLD, thus confirming the applicability of solution-derived BiVO_4_ for fundamental studies of transport mechanisms.

## Conclusions

We introduce a new solution-based synthesis method for wafer-scale epitaxial, monoclinic scheelite BiVO_4_ (010) on YSZ (001) substrates using spin-coating and metalorganic decomposition. The resulting films are compact, planar, and homogeneous with a high structural quality over a complete 2′′ wafer. Indeed, the small rocking curve FWHM of ∼0.04° indicates better structural order than analogous films deposited using much more advanced physical^9–14^ and chemical vapor deposition methods,^16^ and approaching those fabricated by MBE.^15^ This is an excellent result considering that our facile spin-coating process is performed in air at ambient pressure. Importantly, the continuous and compact nature of the films enables precise characterization of optoelectronic characteristics of BiVO_4_, which indicate small Urbach energies, low sub-band gap optical absorption, and thermally activated hopping transport comparable to films produced by PLD. Using these films, we experimentally characterize the large uniaxial optical anisotropy of BiVO_4_, confirming theoretical predictions and indicating a preferred orientation of BiVO_4_ (010) for capturing solar radiation. Thus, the facile spin-coating process for synthesis of epi-BiVO_4_ presented here can be accomplished without complex infrastructure, and enables high structural and optoelectronic quality material that is suitable for a broad range of fundamental studies necessary to advance understanding of the complex processes associated with light-to-chemical energy conversion.

## Conflicts of interest

There are no conflicts to declare.

## Supplementary Material

TA-010-D1TA10732A-s001
