# Supplementary material for: Solution-based synthesis of wafer-scale epitaxial BiVO4 thin films exhibiting high structural and optoelectronic quality
Source: J Mater Chem A Mater. 2022 Apr 22;10(22):12026–34. doi: 10.1039/d1ta10732a (PMC9172877; doi:10.1039/d1ta10732a)
Supplement: TA-010-D1TA10732A-s001 [file TA-010-D1TA10732A-s001.pdf]

Electronic Supplementary Material (ESI) for Journal of Materials Chemistry A.
This journal is © The Royal Society of Chemistry 2022

Supporting Information

Solution-based Synthesis of Wafer-Scale Epitaxial $BiVO_4$ Thin Films Exhibiting High Structural and Optoelectronic Quality

Viktoria F. Kunzelmann,[a] Chang-Ming Jiang,[a] Irina Ihrke,[a] Elise Sirotti,[a] Tim Rieth,[a] Alex Henning,[a] Johanna Eichhorn,[a] and Ian D. Sharp*[a]

[a] Walter Schottky Institute and Physics Department, Technsiche Universität München, Am Coulmobwall 4, 85748 Garching, Germany

*sharp@wsi.tum.de

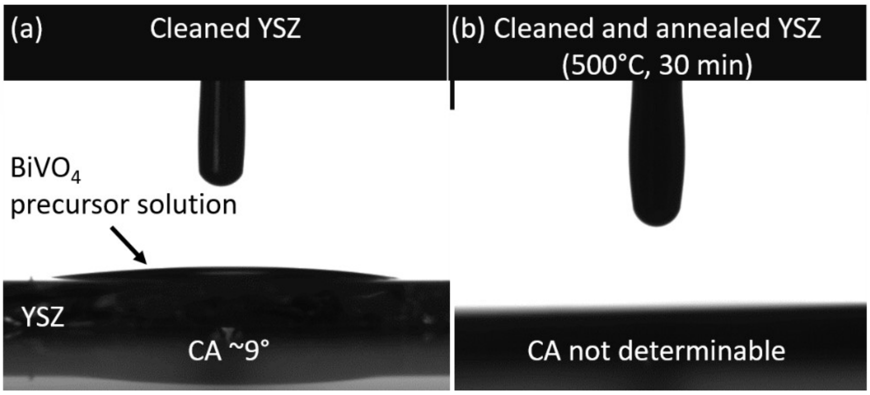

Figure S1. Contact angle of $BiVO_4$ precursor solution on YSZ substrate. Contact angle image acquired (a) on cleaned YSZ and (b) on cleaned and pre-annealed (500 °C for 30 min) YSZ.

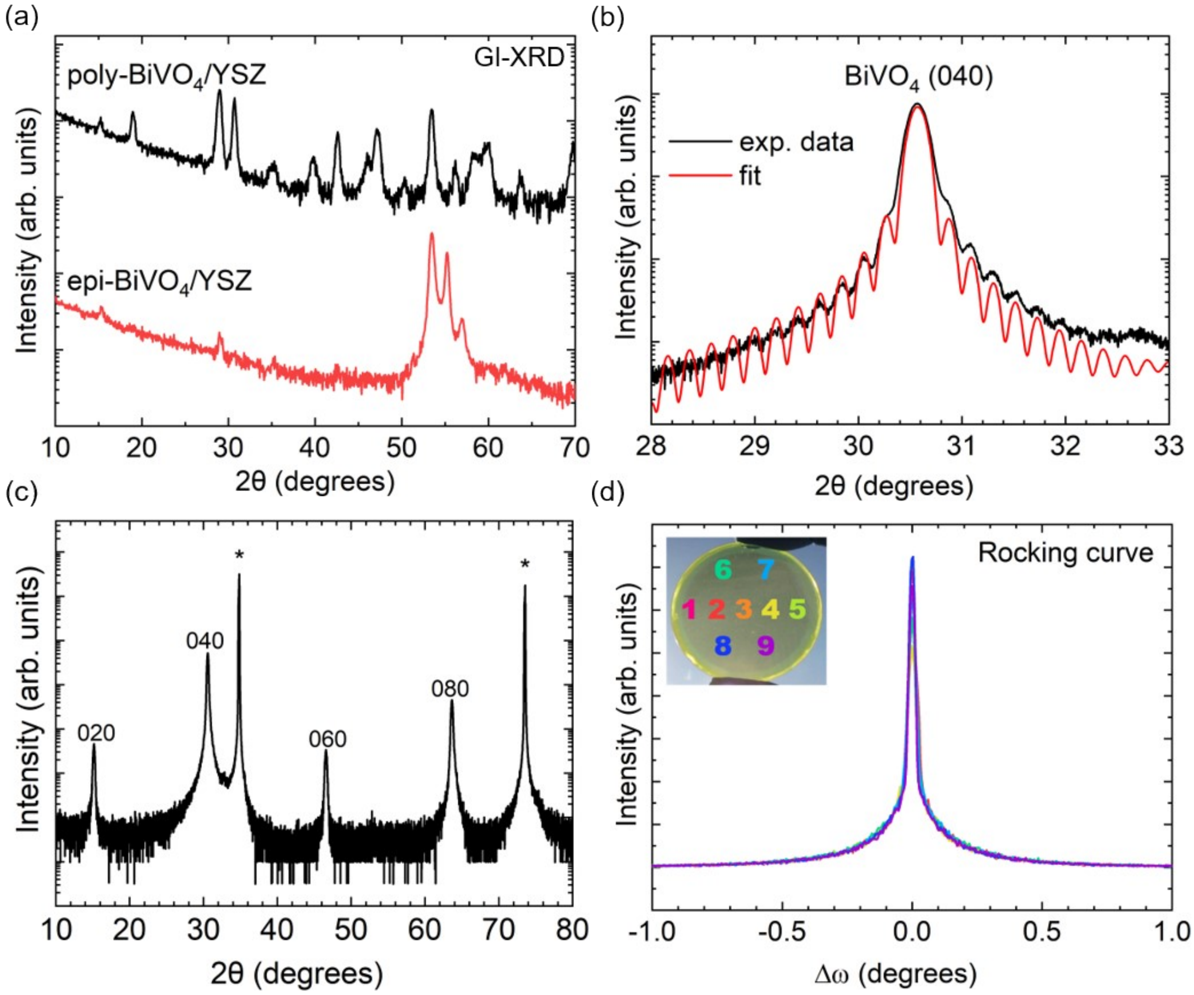

Figure S2. X-ray diffraction characterization of epi- and poly-$BiVO_4$/YSZ. (a) Grazing-incidence XRD (GI-XRD) scans of poly-$BiVO_4$/YSZ and epi-$BiVO_4$/YSZ. Several peaks can be seen between 53° and 58° in the GI-XRD pattern of epi-$BiVO_4$/YSZ. These peaks do not stem from the polycrystalline $BiVO_4$ domains, but instead from the YSZ (113) planes (2θ = 59.48°) as well as from the $BiVO_4$ (061) and (261) planes (2θ = 53.23° and 53.32°, respectively), of which the incline angles make them coincidently detectable in GI-XRD geometry. (b) Pseudo-Voigt fit of the interference oscillations around the $BiVO_4$ (040) peak in the θ-2θ high-resolution XRD (HR-XRD) scan of epi-$BiVO_4$/YSZ for film thickness determination. (c) θ-2θ HR-XRD scan of epi-$BiVO_4$/YSZ with the stars indicating diffraction peaks resulting from YSZ substrate. (d) Rocking curves of epi-$BiVO_4$/YSZ with FWHMs ranging from 0.035° to 0.05° recorded around the $BiVO_4$ (040) peak at 2θ = 30.55° at different measurements positions across the 2” diameter wafer.

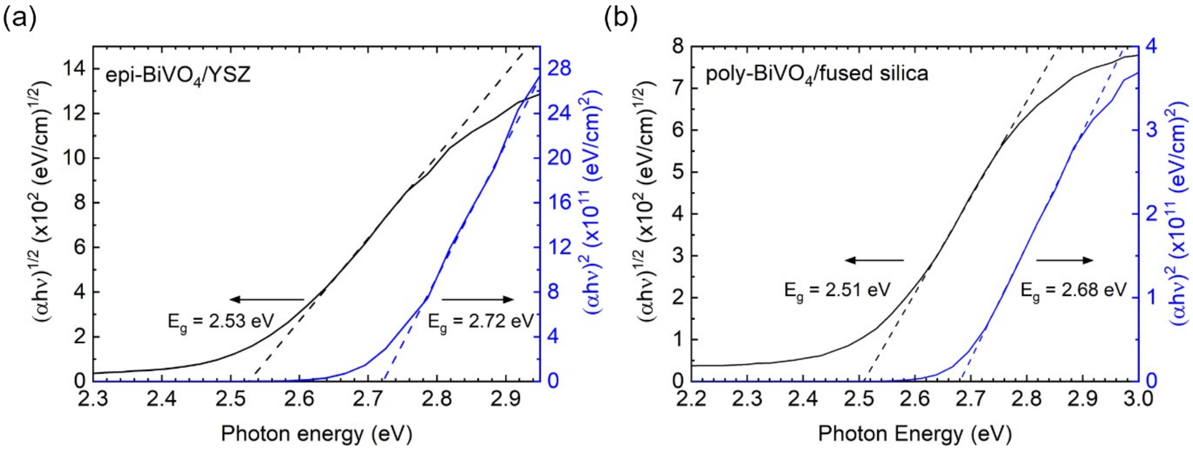

Figure S3. Band gap analysis of epi-$BiVO_4$/YSZ and poly-$BiVO_4$/fused silica. Tauc plots of the PDS absorption data to determine the indirect band gap (black) and the direct transition (blue) of (a) epi-$BiVO_4$/YSZ and (b) poly-$BiVO_4$/fused silica.

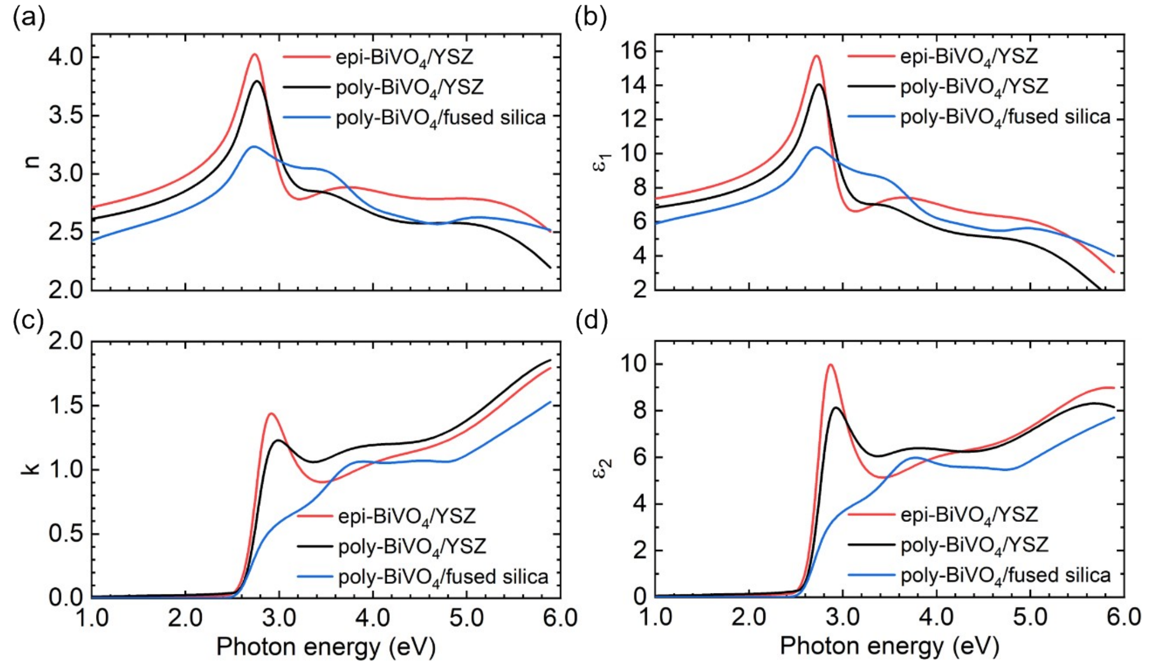

Figure S4. Optical constants of the $BiVO_4$ thin films. (a) The refractive index, *n* (top) the extinction coefficient, *κ* (bottom), as well as (b) the real part ε_1 (top) and the imaginary part ε_2 (bottom) of the dielectric function of epi- and poly-$BiVO_4$/YSZ, and of poly-$BiVO_4$/fused silica obtained by fitting of spectroscopic ellipsometry data.

We note that two different unit cell definitions, which differ in the chosen symmetry and axis assignment, are commonly used for ms-$BiVO_4$. The first convention is to choose the *b*-axis of the unit cell as the unique axis with the space group $C_{2/c}$, and the second is to define the *c*-axis as the unique axis with the space group $I_{2/b}$ (Fig. S1). We will use the first convention, in which the long *b*-axis is given by *b* = 11.697 Å, while *a* = 7.247 Å, and *c* = 5.090 Å, with β = 134.225°[1].

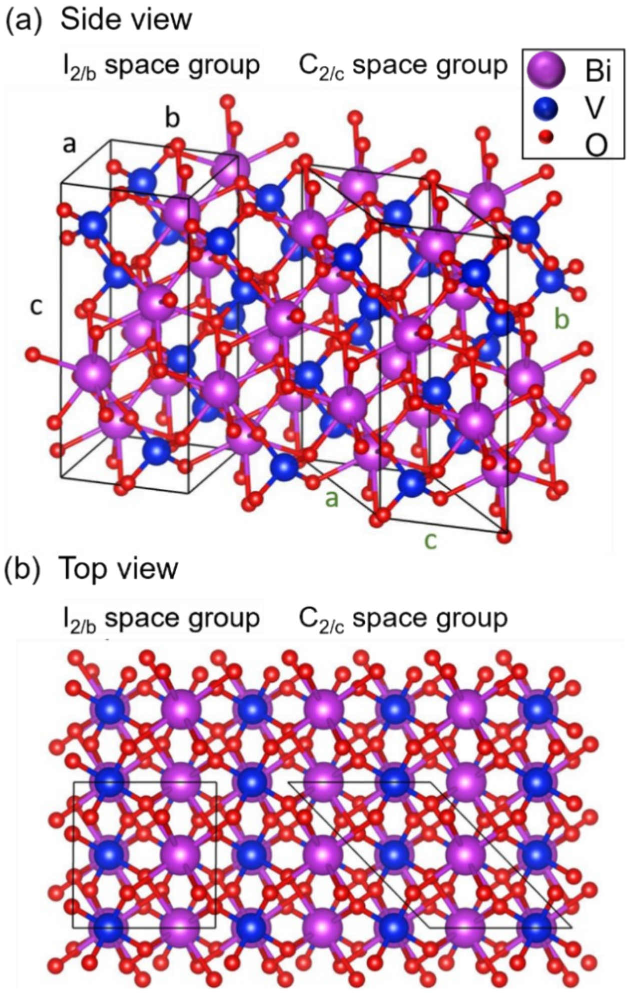

Figure S5. Crystal lattice of monoclinic scheelite $BiVO_4$. Two different conventionally used unit cells are indicated by black lines.

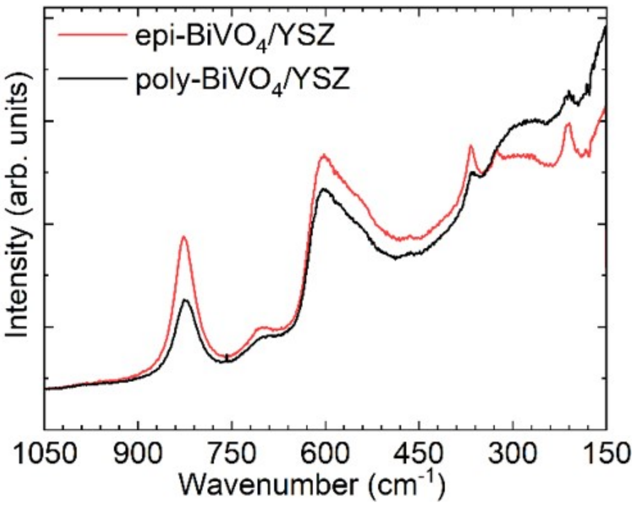

Figure S6. Raman spectra of epi- and poly-$BiVO_4$/YSZ. The spectra of epi- and poly-$BiVO_4$/YSZ thin films confirm the monoclinic scheelite $BiVO_4$ phase, characterized by the $(VO4)^{3-}$ v_1 symmetric stretching mode at ~826.5 cm^{-1}, the v_2 bending modes of the VO_4 units at ~324.4 cm^{-1} and 367.6 cm^{-1}, and the external mode at ~211.5 cm^{-1} comparable to the findings of Frost *et al.*[2]. The strong peak at ~607.8 cm^{-1} can be assigned to the characteristic YSZ F_{2g} peak[3].

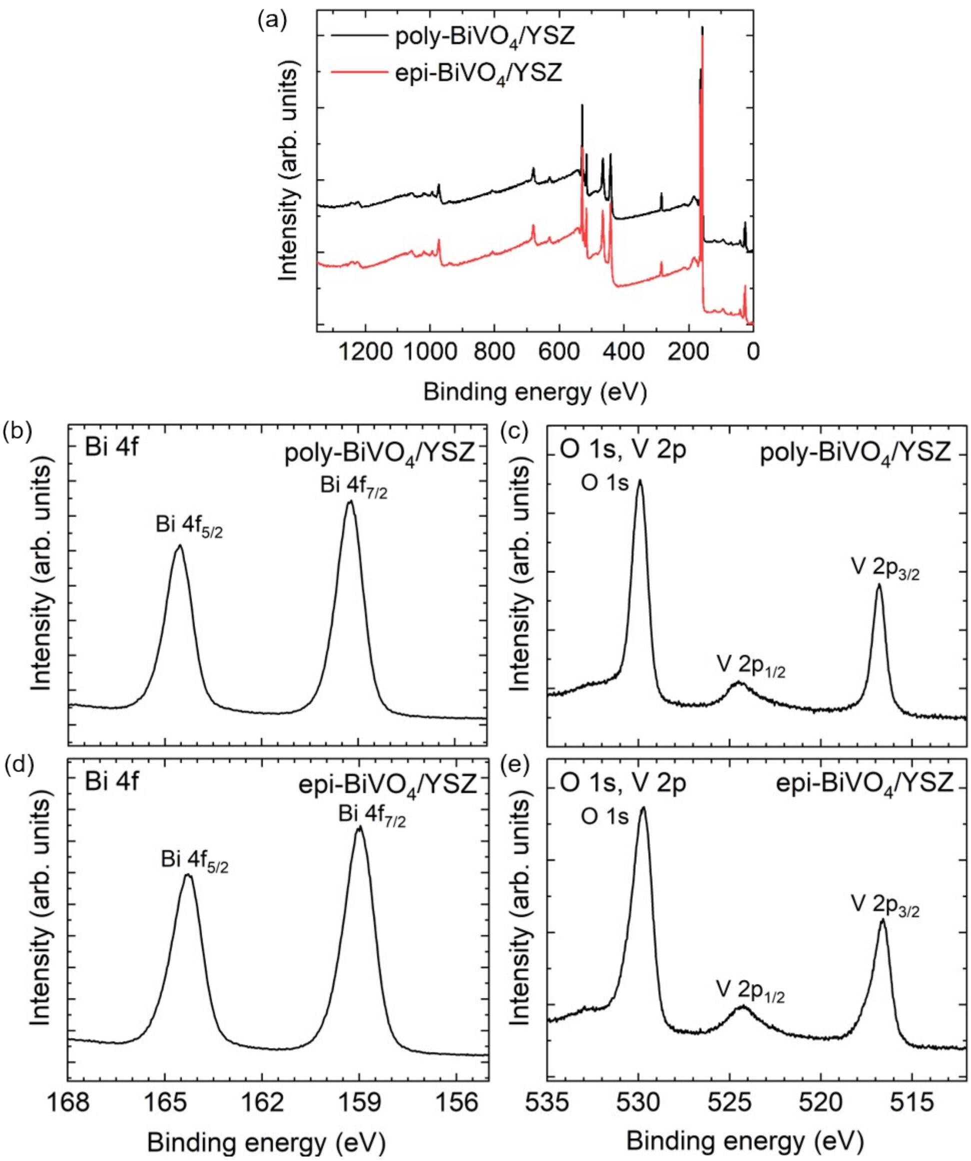

Figure S7. X-ray photoelectron spectroscopy scans of poly- and epi-$BiVO_4$/YSZ. (a) Survey spectra, (b) and (d) Bi 4f, and (c) and (e) O 1s, V 2p high-resolution spectra. The XPS measurements confirm the growth of closed and pure $BiVO_4$ films on YSZ with no detectable impurities and no exposed YSZ substrate. The survey and high-resolution spectra of the epi-$BiVO_4$/YSZ are comparable to those of poly-$BiVO_4$/fused silica in previous studies[4]. The Bi:V ratio for both the poly- and epi-$BiVO_4$/YSZ is ~1.7, indicating a Bi-rich surface as reported in literature for spin-coated polycrystalline $BiVO_4$[4] and no significant change in the surface stoichiometry after the solid-state transformation to epitaxial $BiVO_4$. The Bi-rich surface points to the devolatilization of V during the spin-coating synthesis at higher temperatures in air as previously reported by Lamers *et al.*[5].

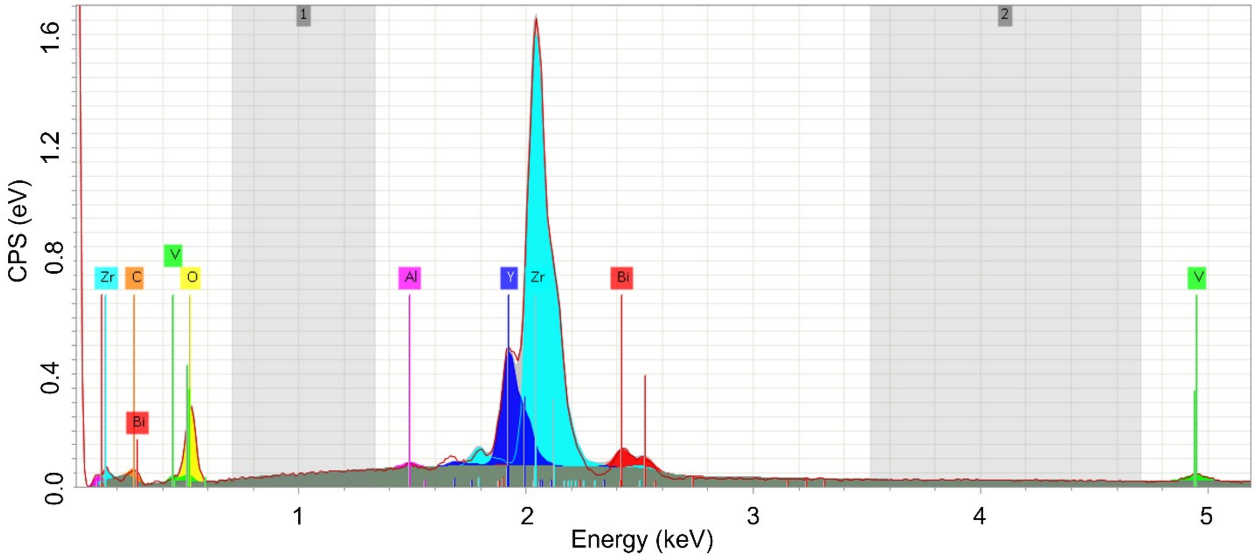

Figure S8. Energy-dispersive X-ray spectrum of epitaxial $BiVO_4$/YSZ with deconvoluted peaks of the individual elements highlighted in color, and the fitted background shown in grey.

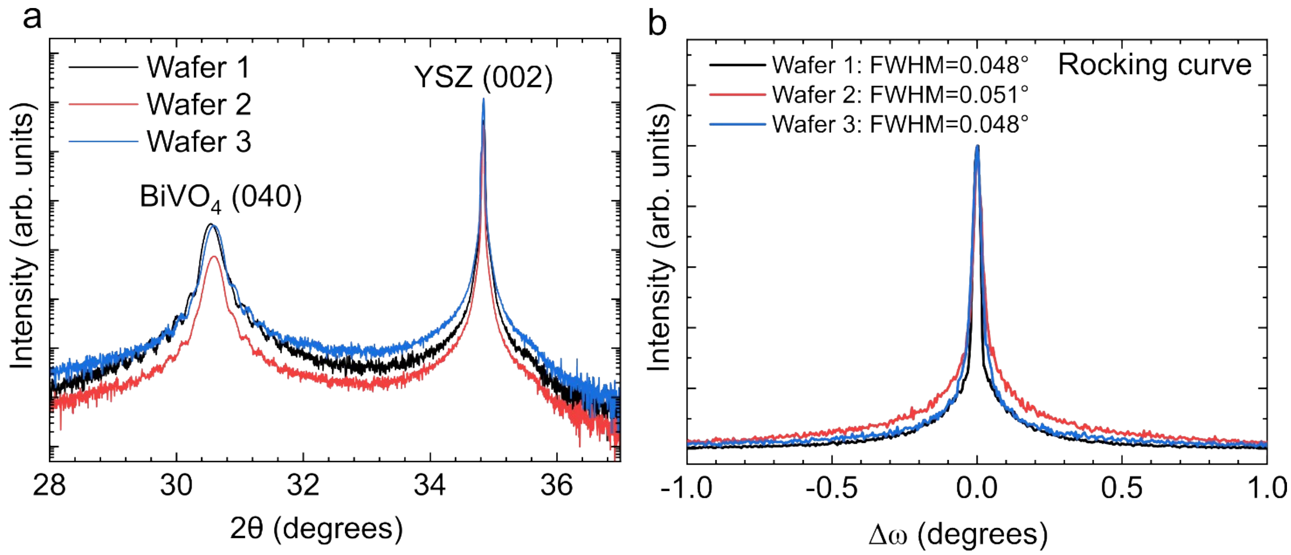

Figure S9. (a) High-resolution XRD θ-2θ scans of epitaxial $BiVO_4$ grown on three different YSZ wafers on different days. (b) Rocking curves of the three different wafers recorded around the $BiVO_4$ (040) peak at 2θ = 30.55°. The corresponding values for the FWHM are given in the plot legend.

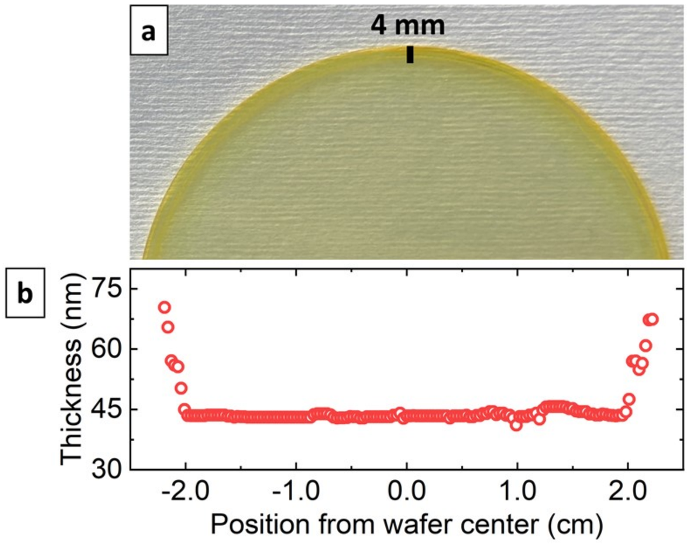

Figure S10. (a) Photograph of an epitaxial $BiVO_4$/YSZ wafer and (b) the thickness of the $BiVO_4$ layer measured along a straight line through the wafer center and obtained by fitting ellipsometry data. Commercial focusing optics to obtain a spot size of 200 μm were used for acquiring the ellipsometry data.

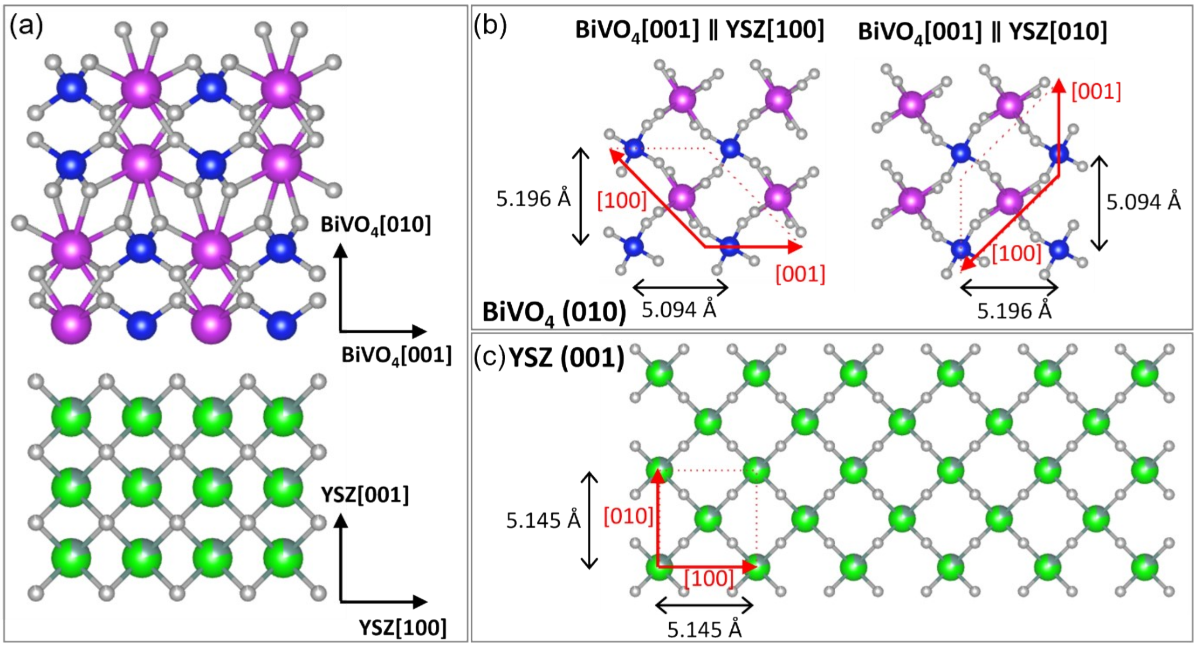

Figure S11. Atomic lattice models of $BiVO_4$/YSZ. (a) $BiVO_4$ (010) on YSZ (001) in side view, and (b) and (c) the in-plane atomic arrangement in top view of $BiVO_4$ [001] ∥ YSZ [100], $BiVO_4$ [001] ∥ YSZ [010] and of YSZ (001), respectively. Bi atoms are indicated in purple, V atoms in blue, O atoms in grey and Zr atoms in green.

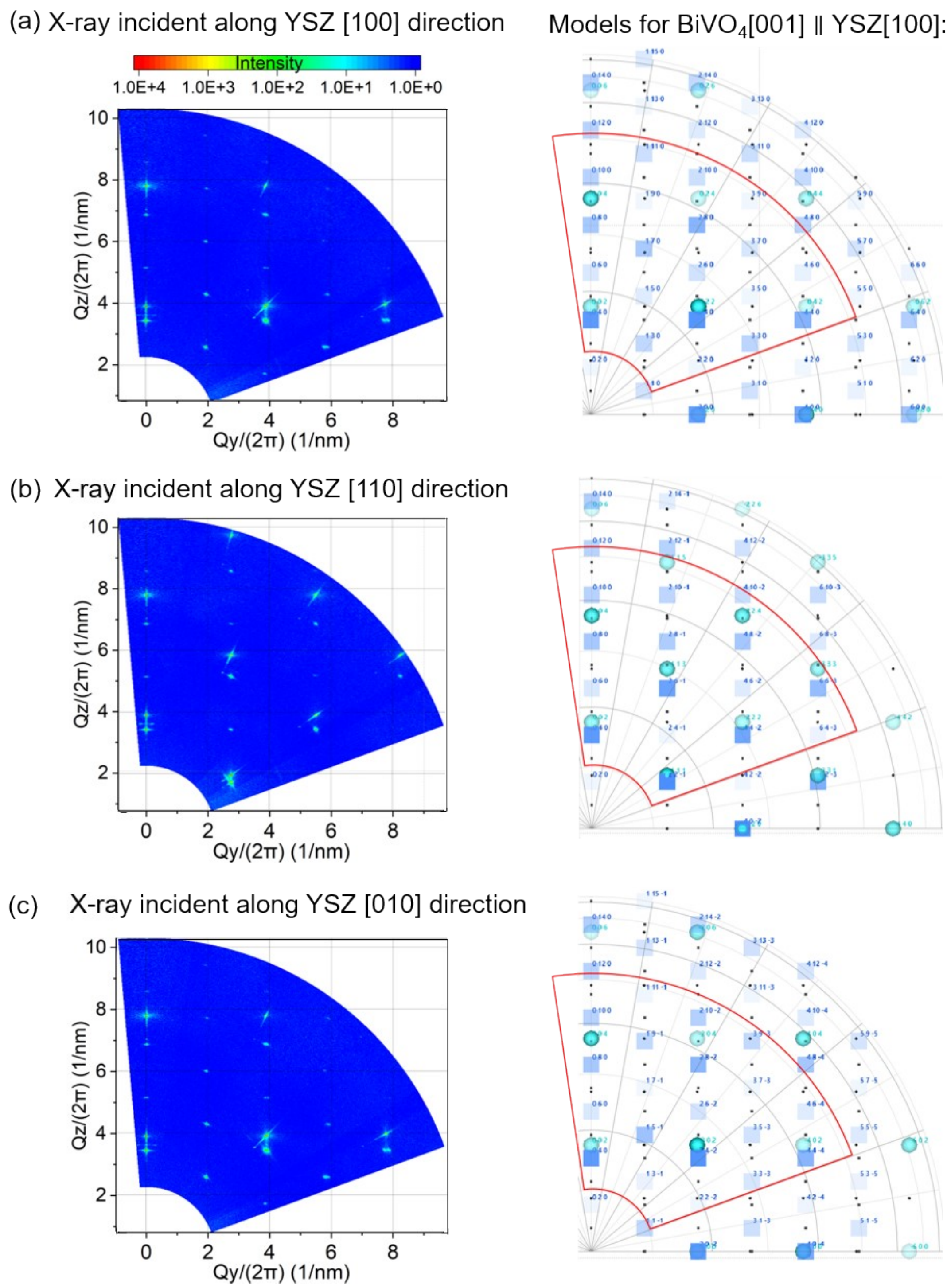

Figure S12. 2D reciprocal space maps (RSMs) of epi-$BiVO_4$/YSZ. X-ray incidence along the YSZ (a) [100], (b) [110], and (c) [010] direction. The corresponding RSMs predicted by the model for $BiVO_4$ [001] ∥ YSZ [100] are shown in the right column.

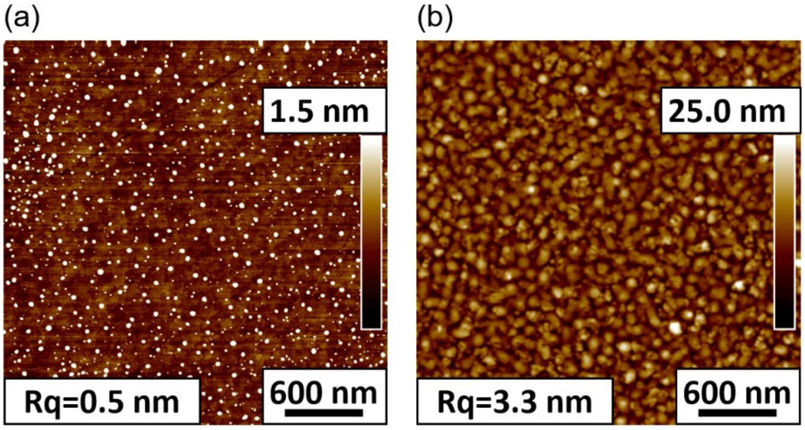

Figure S13. AFM topography images of one layer of spin-coated $BiVO_4$ on pre-treated YSZ after different pyrolysis conditions. AFM image (a) after the pyrolysis directly performed at 650 °C for 10 min and (b) after the pyrolysis performed at the conditions optimized for polycrystalline material (500 °C for 10 min).

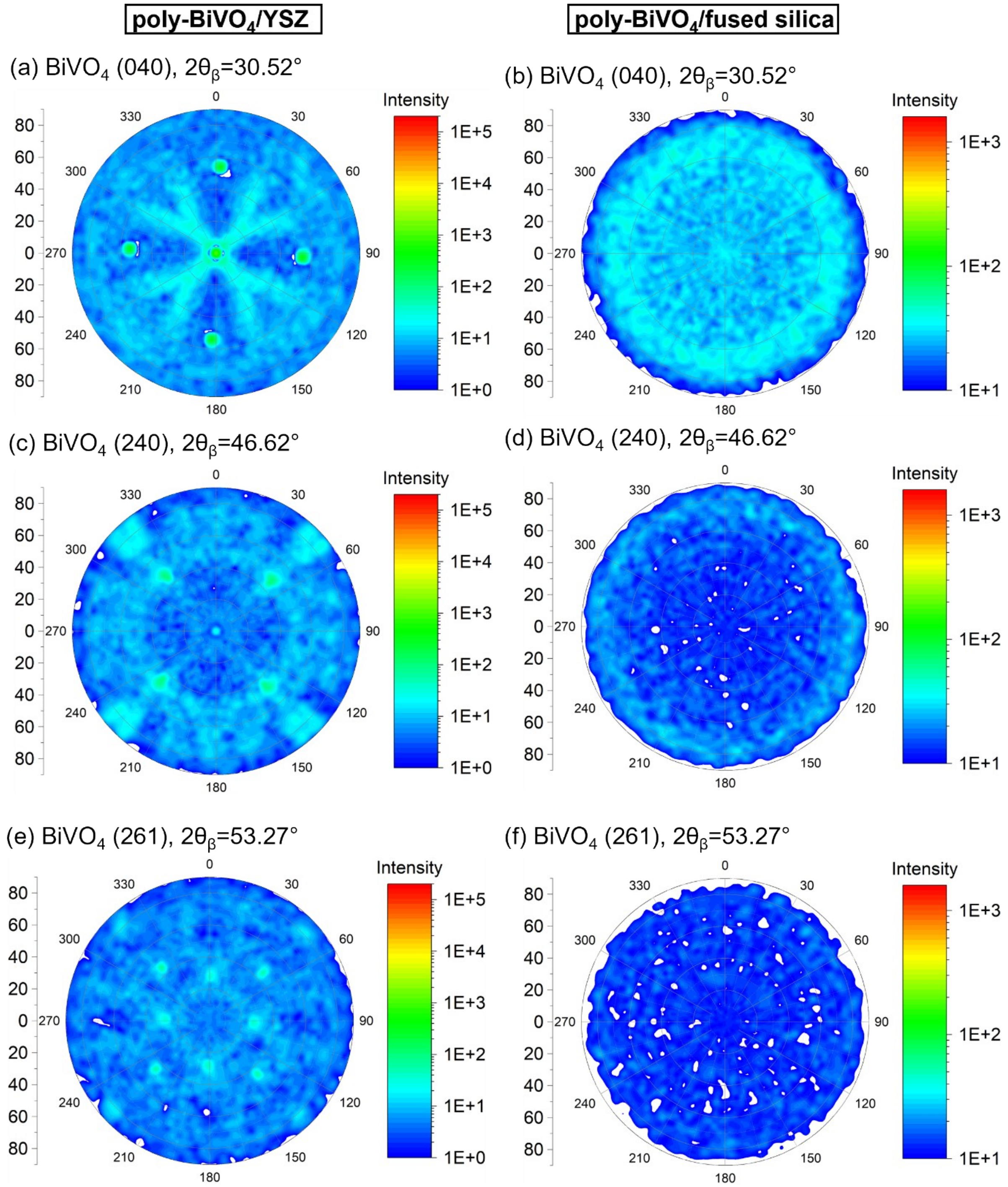

Figure S14. Pole figures of poly-BiVO$_4$ on different substrates. Pole figures of (a, c, e) poly-BiVO$_4$/YSZ and (b, d, f) poly-BiVO$_4$/fused silica.

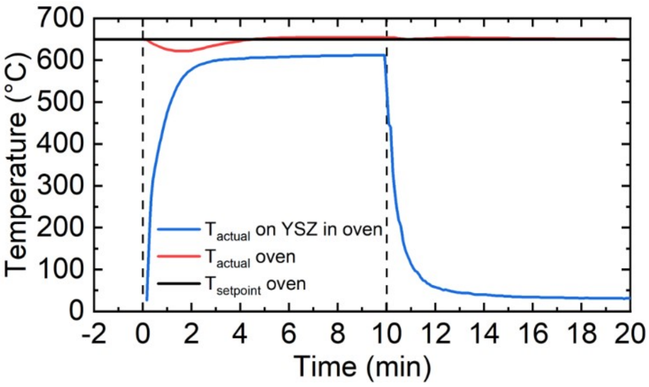

Figure S15. Temperature behavior inside the muffle furnace during the solid-state transformation from poly- to epi-$BiVO_4$/YSZ. Actual temperature (blue) on YSZ inside the muffle furnace and actual temperature of the oven (red) over time with a programmed temperature setpoint of 650 °C (black). At t = 0 min the oven door was opened and the sample was placed inside. After 10 min the sample was taken out (vertical dashed lines). After placing the YSZ substrate inside the furnace preheated to 650 °C at *t* = 0, two different heating ramps can be distinguished in the first 100 s. For the first ~15 s, the highest thermal ramp rate of ~20 °C/s occurs up to ~325 °C. Within the following ~85 s, 90 % of the total temperature increase is reached with a heating ramp of ~3 °C/s. The remaining time the YSZ substrate heats up with significantly decreasing rate until it is taken out of the oven after 10 min.

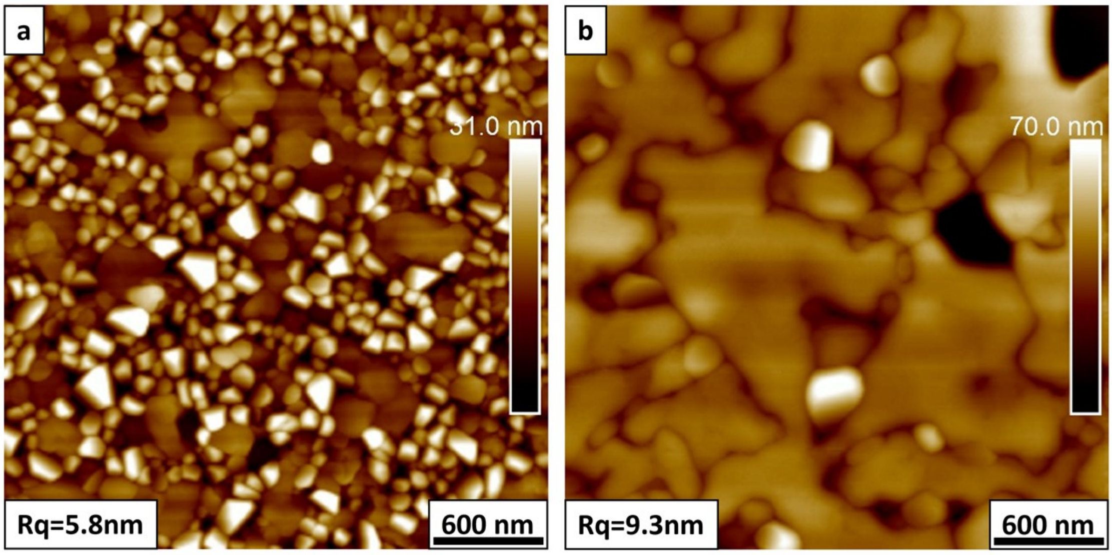

Figure S16. AFM images of $BiVO_4$/YSZ after annealing in a silicon housing for 10 min at (a) 625 °C and (b) at 675 °C.

References

1 A. W. Sleight, H. -y. Chen, A. Ferretti and D. E. Cox, *Mater. Res. Bull.*, 1979, **14**, 1571–1581.
2 R. L. Frost, D. A. Henry, M. L. Weier and W. Martens, *J. Raman Spectrosc.*, 2006, **37**, 722–732.
3 M. B. Pomfret, C. Stoltz, B. Varughese and R. A. Walker, *Anal. Chem.*, 2005, **77**, 1791–1795.
4 F. M. Toma, J. K. Cooper, V. Kunzelmann, M. T. McDowell, J. Yu, D. M. Larson, N. J. Borys, C. Abelyan, J. W. Beeman, K. M. Yu, J. Yang, L. Chen, M. R. Shaner, J. Spurgeon, F. A. Houle, K. A. Persson and I. D. Sharp, *Nat. Commun.*, 2016, **7**, 1–11.
5 M. Lamers, S. Fiechter, D. Friedrich, F. F. Abdi and R. van de Krol, *J. Mater. Chem. A*, 2018, **6**, 18694–18700.
